# Optimization of the empirical antibiotic choice during the treatment of acute prosthetic joint infections: a retrospective analysis of 91 patients

**DOI:** 10.1080/17453674.2019.1621595

**Published:** 2019-05-28

**Authors:** Joost H J Van Erp, Adriaan C Heineken, Remco J A Van Wensen, Robin W T M Van Kempen, Johannes G E Hendriks, Marjolijn Wegdam-Blans, Judith M Fonville, M C (Marieke) Van Der Steen

**Affiliations:** a Department of Orthopaedic Surgery, Catharina Hospital Eindhoven, Eindhoven;; b Orthopaedic Center Máxima, Máxima Medical Center, Eindhoven;; c Laboratory of Medical Microbiology, Stichting PAMM, Veldhoven, The Netherlands

## Abstract

Background and purpose — The preferred treatment of an acute prosthetic joint infection (PJI) is debridement, antibiotics, irrigation and retention of the prosthesis (DAIR). The antibiotic treatment consists of an empirical and targeted phase. In the empirical phase, intravenous antibiotics are started after surgery before micro-organisms are determined in microbiological cultures. Which empirical antibiotic is used differs between hospitals, partly reflecting geographic differences in susceptibility spectrums. We investigated whether flucloxacillin should remain the antibiotic of choice in our hospital for empiric treatment of acute PJI with DAIR.

Patients and methods — We retrospectively analyzed 91 patients treated for PJI with DAIR between 2012 and 2016. The susceptibility of micro-organisms was determined in multiple cultures of periprosthetic tissue and synovial fluid for 3 antibiotics: amoxicillin/clavulanic acid, cefazolin, and flucloxacillin.

Results — Positive microbiological cultures from 68 patients were analyzed. *Staphylococcus aureus* was the predominant pathogen, cultured in half of the patients. In one-third of patients more than 1 micro-organism was found. On a patient level, the data showed that 65% were responsive to flucloxacillin, 76% to amoxicillin/clavulanic acid, and 79% to cefazolin.

Interpretation — Flucloxacillin appeared to be a suboptimal choice in our patient population treated with DAIR. We therefore changed our practice to cefazolin as the preferred antibiotic in the empirical treatment of acute PJI with DAIR.

A prosthetic joint infection (PJI) typically develops in 1 of 3 ways: through perioperative colonization of the implant, hematogenous seeding caused by a bacteremia, or spread from an infection of the surrounding tissue (Widmer 2001). Furthermore, PJI can be classified in 3 time categories. Early infections occur within 3 months after implantation. Delayed PJI appears 3–24 months after implantation and late PJI after 24 months (Zimmerli et al. 2004). Early and hematogenous PJIs are classified as acute infections, which often have an acute onset and are caused by virulent micro-organisms (Zimmerli et al. 2004).

The recommended treatment of an acute PJI is drainage, antibiotics, irrigation, and retention of the prosthesis (DAIR) (Zimmerli et al. 2004). DAIR, for hip and knee prostheses, has a success rate of approximately 70% (Kuiper et al. 2014). In the empirical phase intravenous antibiotics are started blind after surgery until the causative micro-organisms are determined in microbiological cultures. The importance of tailored antibiotic treatment during the targeted phase is well known (Argenson et al. 2019). However, far less literature is available on which antibiotic to use in the empirical phase. The most frequently cultured micro-organisms in PJI are coagulase-negative staphylococci and *Staphylococcus aureus* (Phillips et al. 2006, Stefánsdóttir et al. 2009, de Vries et al. 2016). Other commonly found micro-organisms are streptococci, gram-negative bacilli, enterococci, and anaerobes (Segawa et al. 1999, Steckelberg and Osmon 2000). In approximately 46% of acute PJI multiple pathogens are found in a patient (de Vries et al. 2016).

Reflecting local policy, micro-organism prevalence, and resistance patterns, the antibiotics used in the empirical phase differ between countries and hospitals (Kuiper et al. 2016). Moran et al. (2007) (in the United Kingdom) advised a combination of vancomycin and carbapenem, both broad-spectrum antibiotics, as empirical antibiotic regime during DAIR. The population in the UK consisted of a relatively high number of patients with methicillin-resistant *Staphylococcus aureus* (MRSA) or cephalosporin- and beta-lactam resistance. Sousa et al. (2010) (in Portugal) found similar results and recommended the same regime. Since MRSA prevalence in the Netherlands is much lower than in those countries (van Cleef et al. 2013), double therapy with broad-spectrum antibiotics might not be in line with good antibiotic stewardship (Tiemersma et al. 2012). In our orthopedic infection center, flucloxacillin has been the antibiotic of first choice in the empirical phase, because of its high effectiveness against common pathogens like *Staphylococcus aureus* and coagulase-negative staphylococci.

Ideally, the empiric antibiotic treats as many patients as possible, has limited side effects and no restricted usage. Optimal empiric antibiotic therapy contributes to an effective treatment of PJI and will result in better clinical outcomes regarding retention of the prosthesis, complications, and morbidity. We determined which antibiotic should be used in the empirical phase of the treatment of acute PJI with DAIR, based on analyses of local culturing results on PJI samples. We focus on 3 commonly used antibiotics, namely amoxicillin/clavulanic acid, cefazolin, and flucloxacillin.

## Patients and methods

Using electronic medical records, we retrospectively identified eligible patients from the Catharina Hospital in Eindhoven based on Dutch diagnosis treatment codes for irrigation of the knee or hip joint (CoTG 038640 or 038540, respectively). Patients who were diagnosed with a PJI and underwent DAIR are registered under these codes. We included patients who were diagnosed with a PJI and had a DAIR performed between 2012 and 2016. Additional inclusion criteria entailed a joint prosthesis in situ and the suspicion of PJI within 90 days of index surgery or in case of a hematogenous infection within 3 weeks after onset of PJI signs. Furthermore, only patients from whom periprosthetic tissue was obtained during surgery and sent for microbiological analysis were included. Patients with negative cultures were excluded from the analyses. Patients who underwent DAIR between 2014 and 2016 in the Máxima Medical Center, which was collaborating with the same microbiological laboratory, were included following the same criteria.

### Prevention and treatment and of PJI

To prevent PJI, several recommended measures have been taken in both centers. We swab preoperatively for nasal carriage of *S. aureus*. When the results are positive for *S. aureus*, patients use chlorhexidine scrub and Bactroban (mupirocin). Cefazolin is used as prophylactic antibiotic, during primary and revision arthroplasty. All DAIR were performed according to the regional treatment protocol for PJI. Part of the DAIR treatment is taking multiple cultures of periprosthetic tissue and synovial fluid before perioperative prophylaxis is administered (Zimmerli et al. 2004). For collection of the cultures, separate clean instruments were used. Typically, 5 cultures per patient were obtained. After debridement, interchangeable parts are replaced and the joint is flushed with at least 3L of saline, using pulsed lavage.  

### Microbiological analyses

Following local protocol, samples were incubated on aerobic and anaerobic agar plates and plates were examined for bacterial growth after 2, 7, and 14 days. In the case of bacterial growth, all colony-forming units were determined with MALDI. After determination of the micro-organism(s), antimicrobial resistance was measured using VITEK (BioMérieux, Marcy-l’Étoile, France). For each patient, multiple samples were sent for culturing. If a micro-organism was found in only 1 of the samples, it was excluded from analysis as it was considered contamination. An exception was made for *Staphylococcus aureus*, as infection with this organism could possibly have such grave consequences that the risk of missing this infectious agent theoretically outweighed the risk of treating contamination.

After routine laboratory antibiotic susceptibility testing of the micro-organisms as described above, the measured susceptibility patterns were supplemented with known intrinsic and derived resistance information as described by EUCAST (Leclercq et al. 2013). The resistance pattern for cefazolin was equated to the resistance of cefuroxime. Resistance was further inferred from related antibiotics and literature studies as follows. *Corynebacterium, Finegoldia, Granulicatella, Peptoniphilus, Cutibacterium acnes*, and streptococcal species were set to be sensitive to cefuroxime when sensitive to penicillin. Streptococci and *Granulicatella* species were set to be sensitive to amoxicillin/clavulanic acid when sensitive to penicillin, whilst *Enterococcus faecium* was set to be resistant to amoxicillin/clavulanic acid. For some combinations of micro-organism and antibiotic, the resistance pattern could not be inferred from known patterns or literature studies. Susceptibility was then set as unknown.

### Analyses

The measured and inferred susceptibility patterns were used to determine susceptibility to the different antibiotics, on the levels of both micro-organism and patient. A patient was considered responsive if all cultured micro-organisms were reported as sensitive to the antibiotic.

For statistical analysis the McNemar test was used to compare the sensitivity of the evaluated antibiotics cefazolin and amoxicillin/clavulanic acid against flucloxacillin.

### Ethics, funding, and potential conflicts of interest

The Medical Research Ethics Committees United declared that this study did not meet the criteria as stated by the Medical Research Involving Human Subjects Acts (WMO) and the local committee approved this retrospective cohort study (nWMO-2017.51). No funding was received for this study. There are no potential conflicts of interest.

## Results

### Patients

We analyzed available data for the 91 patients who presented themselves with a suspicion of PJI and subsequently underwent DAIR (Table 1). A median of 4 (1–8) cultures were acquired per patient. In 14 patients no micro-organisms could be cultured, despite clinical signs of PJI. Another 9 patients were excluded because only 1 positive culture was found, which was considered contamination (except when *Staphylococcus aureus* was found (n = 4). Culturing results of the remaining 68 patients were included in the analyses.

**Table 1.** Patients’ characteristics. Values are number unless otherwise specified

Factor	N = 91
Male sex	54
Age at time of DAIR, mean (SD)	73 (10)
ASA classification	
1	4
2	52
3	31
4	1
Unknown	3
Type of index arthroplasty	
Total knee arthroplasty	37
Total hip arthroplasty	33
Hemi-arthroplasty	13
Revision total hip arthroplasty	8
Type of acute PJI	
Early	75
Hematogenous	16
Months after index surgery, mean (SD)	
Early	1 (18)
Hematogenous	65 (53)

DAIR: debridement, antibiotics, irrigation, and retention of the prosthesis;

ASA: American Society of Anesthesiologists’ classification of Physical Health

### Microbiological analyses

In 43 of the 68 patients who had positive microbiological cultures, only a single micro-organism was found, while in 25 cases of acute PJI multiple micro-organisms were found in 1 patient (there were 18 patients with 2 organisms; 5 patients with 3 organisms, 1 patient with 4 organisms, and 1 patient with 5 organisms). 31 different micro-organisms were determined, 16 of which were found in only a single patient. Micro-organisms were grouped in commonly used relevant biological categories. Categories that were found only in a single patient were collated in the group ‘other’, which consists of *Finegoldia magna, Granulicatella adiacens, Mycoplasma hominis, Peptoniphilus harei, Acinetobacter genomospecies,* and *Cutibacterium acnes*. The most commonly found group of micro-organisms was *Staphylococcus aureus* (34 patients). Other frequently cultured micro-organisms were other coagulase-negative staphylococci, found in 13 patients, and non-group A/B hemolytic streptococci, determined in 11 patients. In none of the patients were multi-resistant organisms found.

### Antibiotic susceptibility

#### Micro-organisms

**Figure 1.**
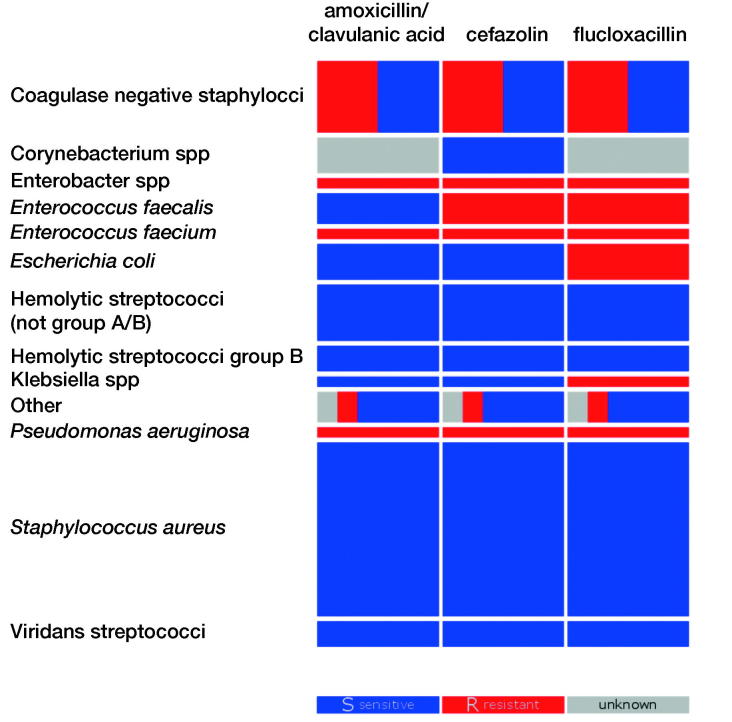
Sensitivity of the most common groups of micro-organisms. Sensitive (S) is displayed in blue, resistant (R) in red, and unknown in grey. The blocks’ height represents the prevalence of the micro-organism. The coagulase-negative staphylococci exclude *Staphylococcus aureus*; the group “other” consists of *Finegoldia magna, Granulicatella adiacens, Mycoplasma hominis, Peptoniphilus harei, Acinetobacter genomospecies*, and *Cutibacterium acnes*.

For each cultured micro-organism, the sensitivity to the 3 evaluated empiric antibiotic options was determined based on measured and inferred resistance patterns, for each culture in each patient separately (Figure 1).

#### Effectiveness for each patient

Patients can be infected by more than a single micro-organism. For treatment choice it is therefore important to evaluate how many patients would have been treated effectively with each of the different antibiotics in the empirical phase of PJI infection with DAIR.

For some micro-organisms, the sensitivity to antibiotics was not measured or inferred. We evaluated patients with such micro-organisms as either sensitive or resistant in 2 separate analyses. In both scenarios, a substantially lower sensitivity for flucloxacillin, the currently used empiric antibiotic in our clinic, was seen (Table 2). Conservatively interpreting unknown as resistant, we conclude that patients would have responded better to cefazolin (p = 0.002) or amoxicillin/clavulanic acid (p = 0.008) than the currently used flucloxacillin. 

**Table 2.** Sensitivity of used antibiotics in percentages with 95% confidence interval based on the binomial distribution

Antibiotic	%patients sensitive	
	(unknown = resistant)	(unknown = sensitive)
Amoxicillin/clavulanic acid	77 (65–86)	84 (73–92)
Cefazolin	79 (68–88)	81 (70–89)
Flucloxacillin	65 (52–76)	72 (60–82)

## Discussion

We gained improved insight into the prevalence of different micro-organisms with their resistance pattern to tailor our treatment protocol. The optimal treatment of an acute PJI is DAIR, in which an empiric antibiotic effective against the most commonly found micro-organisms is essential. The variety of micro-organisms and susceptibility patterns in our population, and as a result which antibiotic would perform best, were unknown prior to this study. Our results show that in one-third of the patients, multiple micro-organisms were found in each patient, which emphasizes the importance of a broad-spectrum antibiotic (Moran et al. 2007). Recent studies confirmed that a higher effectiveness of antibiotics is related to fewer failures and better results in long-term follow-up (Puhto et al. 2015).

Comparing 3 antibiotics used in the Netherlands for empiric therapy, we conclude that patients would be more sensitive to treatment with cefazolin and amoxicillin/clavulanic acid than flucloxacillin. Prior to this study, the preferred empiric antibiotic for PJI in our center was flucloxacillin. The results of this study precipitated a change in protocol to cefazolin as antibiotic in the empirical phase. Another option was amoxicillin/clavulanic acid, the effectiveness of which is comparable to cefazolin. There were 2 reasons to prefer cefazolin to amoxicillin/clavulanic acid. First, cefazolin has few side effects and is widely used as a prophylactic for surgery (Bratzler et al. 2013). Second, allergic reactions are thought to occur more frequently to amoxicillin/clavulanic acid than to cefazolin. In our study, no patients had reported allergies to cefazolin, while eight patients were allergic to amoxicillin/clavulanic acid.

The prevalence of the micro-organisms found in our study, with a high number of patients infected with *Staphylococcus aureus* or coagulase negative staphylococci, is in line with other reports (Phillips et al. 2006, Stefánsdóttir et al. 2009, de Vries et al. 2016). However, recent studies, performed in northern China and the USA, demonstrated geographical differences in susceptibility spectrums (Ravi et al. 2016, Li et al. 2018). These studies investigated similar groups of patients, but reported a completely different prevalence of micro-organisms and, with this, other empiric antibiotics were recommended. Also compared with Moran et al. (2007) and Sousa et al. (2010), we found less resistant microorganisms, which enables us to use cefazolin instead of glycopeptides and carbapenem. The latter 2 antibiotics are usually reserved for multidrug-resistant infections and have more side effects, and are therefore less eligible as empiric antibiotics. Furthermore, inappropriate use could result in more resistance. Our findings are in line with a study by Schindler et al. (2013), who were dissuaded from the use of a glycopeptides and carbapenem in the empirical phase because no more failures were seen using only a penicillin or cephalosporin. However, only half of the patients in that study had an infected prosthesis; the other included patients had infected osteosyntheses, such as plates or nails (Schindler et al. 2013). The geographical differences in susceptibility spectrums highlights the importance of locally tailored antibiotic treatment protocols. Our study is the first that evaluated the efficiency of empiric antibiotics in PJI in the Netherlands. The inclusion of patients of 2 medical centers might have affected the cultured micro-organisms. However, we think this bias is limited, since the hospitals are located in the same city, perform comparable surgeries, orthopedic surgeons work in cooperation, follow the same treatment protocol, and treat a similar population.

We included both early postoperative and hematogenous PJIs in our definition of acute PJI patients, which could potentially create a bias. However, we expect that the effect of this bias is limited as both groups were treated with DAIR, and are typically the result of similar micro-organisms. In our centers these patients are subjected to the same PJI treatment protocol. In future studies, subgroup analysis of microbiological cultures from early postoperative versus hematogenous PJI could be conducted; this was not possible in our patient group, because there was only a small group of patients with hematogenous PJI.

In summary, in this study we characterized which micro-organisms caused acute PJI and analyzed their pattern of resistance. With this information we compared the efficiency of three commonly used antibiotics when treating PJI, namely amoxicillin/clavulanic acid, cefazolin, and flucloxacillin. Flucloxacillin was the local empiric antibiotic of choice but proved to be a suboptimal choice in our patient population treated with DAIR. We have changed our practice to the use of cefazolin as the preferred antibiotic in the empirical treatment of acute PJI with DAIR. Optimizing antibiotic therapy could potentially contribute to more effective treatment of PJI and hence logically result in better clinical outcomes and less morbidity. Since susceptibility spectrums differ geographically, it is recommended that centers study their local data to evaluate which antibiotic will optimally treat their PJI patients.
